# Re-evaluating the relationship between missing heritability and the microbiome

**DOI:** 10.1186/s40168-020-00839-4

**Published:** 2020-06-08

**Authors:** Gavin M. Douglas, Joseph P. Bielawski, Morgan G. I. Langille

**Affiliations:** 1 Department of Microbiology and Immunology, Dalhousie University, Halifax, NS Canada; 2 Department of Biology, Dalhousie University, Halifax, NS Canada; 3 Department of Mathematics and Statistics, Dalhousie University, Halifax, NS Canada; 4 Department of Pharmacology, Dalhousie University, Halifax, NS Canada

**Keywords:** Microbiome, Human, Genetics, Environment, Heritability, GWAS, GxE, Hologenome, Holobiont

## Abstract

Human genome-wide association studies (GWASs) have recurrently estimated lower heritability estimates than familial studies. Many explanations have been suggested to explain these lower estimates, including that a substantial proportion of genetic variation and gene-by-environment interactions are unmeasured in typical GWASs. The human microbiome is potentially related to both of these explanations, but it has been more commonly considered as a source of unmeasured genetic variation. In particular, it has recently been argued that the genetic variation within the human microbiome should be included when estimating trait heritability. We outline issues with this argument, which in its strictest form depends on the holobiont model of human-microbiome interactions. Instead, we argue that the microbiome could be leveraged to help control for environmental variation across a population, although that remains to be determined. We discuss potential approaches that could be explored to determine whether integrating microbiome sequencing data into GWASs is useful.

Video abstract

Video abstract

## Background

Genome-wide association studies (GWASs) aim to identify the genetic variants underlying trait heritability. The genetic variance explained by these genetic variants, typically single-nucleotide polymorphisms (SNPs), in a GWAS can be calculated as the combined effect size of all significant genetic variants [1]. For virtually all complex human diseases, this approach explains only a small proportion of the heritability, the proportion of phenotypic variance accounted for by genetic variance in a given population, as inferred from classical heritability studies. For instance, using traditional approaches the heritability of schizophrenia liability was estimated to be 81% [2], but only ~ 3% could be explained based on significant SNPs in a GWAS [3]. Similarly, one traditional estimate for Crohn’s disease liability was 75%, but only 26%, at most, could be explained based on significant SNPs in a GWAS [4]. These examples are two of many recurrent observations that have famously been summarized as “The Case of the Missing Heritability” [5]. Many potential explanations for this missing heritability have been proposed.

The most widely accepted explanation for missing heritability is that GWASs are not testing the majority of relevant human genetic variation [6, 7]. This explanation is supported by the observation that the majority of missing heritability for several traits can be recovered by integrating all genome-wide SNPs into a prediction model [8–10], as compared to using only SNPs individually associated with the phenotype. Similarly, a recent study of heritability for height and body mass index in 21,620 individuals of European ancestry reported that all the expected heritability could be recovered by integrating extremely rare genetic variants in the analysis [11]. These findings suggest that missing heritability may be resolved by accommodating genome-wide sampling of sparsely distributed human genetic variants (largely represented by SNPs) within GWASs.

However, there are other types of segregating genetic variation that could improve GWAS-derived heritability estimates. In particular, copy-number variation, variation in the presence of large genomic regions containing genes, is typically not assessed in GWASs. Ignoring these variants is a major limitation of current GWASs because they represent a substantial proportion of genetic variation: at least 5% of human genomes contain instances of copy-number variation greater than 500 kb [12]. In addition, the pan-genome of 910 individuals of African descent contains ~ 10% more DNA than the human reference genome [13]. Integrating such structural variation, and other unaccounted sources of genetic variance, into GWASs could improve heritability estimates [6].

A related explanation for missing heritability is that many genetic variants have differential effects depending on environmental variation, termed GxE interactions. These interactions, in addition to independent genetic and environmental effects, are known to contribute to the liability of many human diseases [14]. For example, a single nucleotide position in the promoter region of the CD14 gene, which encodes a lipopolysaccharide receptor, is known to interact with several environmental exposures, such as microbial exposure [15, 16]. Different SNPs at this position are associated with increased risk of developing asthma depending on environmental exposures [17]. Without taking these varying environmental exposures into account, the association of each SNP with asthma risk would be underestimated. This example highlights an important limitation of GWASs in cases where GxE interactions influence phenotypes: the genetic variance underlying phenotype variance can be underestimated.

Despite the importance of GxE interactions, genetic and environmental factors are typically analyzed independently. The independent treatment of these factors is mainly due to the prohibitively large sample sizes required for systematically identifying GxE interactions [18]. In addition, there are many challenges facing the reliable estimation of GxE phenotypic effects, including how to assess a representative set of environmental exposures. It has previously been suggested that integrating other “omics” datatypes when detecting GxE interactions might help identify differential exposures across individuals [19, 20]. However, how to best integrate these datatypes into GWASs remains a major challenge [21, 22].

Another explanation for missing heritability is that heritability estimates from classical heritability studies and GWASs may not be directly comparable. GWASs typically estimate narrow-sense heritability, which corresponds to the additive contribution of genetic variants to heritability. In contrast, heritability estimates from classical familial and twin studies are more similar to broad-sense heritability, because they can incorporate non-additive genetic effects [23]. In addition, classical heritability estimates have been criticized due to invalid assumptions regarding shared environmental variation, particularly between monozygotic and dizygotic twins [23, 24].

Despite these issues, a meta-analysis of 2748 twin studies suggested that non-additive genetic effects are unlikely to be substantial contributors to the heritability of human traits [25]. This claim was based on the observation that trait correlations between monozygotic twins were roughly twice as high compared to dizygotic twins, for 84% of the traits analyzed. Importantly, this trend was not true for all studies and traits, but the heritability of human traits was consistent overall with a simple additive genetic model.

It remains controversial whether genetic variants identified through GWASs are the sole contributors to additive genetic variation assessed in classical studies. For instance, it has previously been argued that cultural transmission could be inflating classical estimates of additive genetic effects [26]. Similarly, vertical transmission of epigenetic signals also has been suggested to contribute to classical estimates of additive genetic effects [27, 28]. In this case, some argue that epigenetic signatures should be integrated into GWAS heritability estimates [28], analogously to integrating additional rare genetic variants. Although this is still disputed [29], this example highlights that traditional definitions for valid contributors to additive genetic effects might be overly restrictive [30].

## Enter the human microbiome

The human microbiome refers to the microbes living on and within the body and the functions they encode. These microbial functions include roughly 100 times more genes than the human genome [31]. Whether these microbial genes should be viewed as an additional source of human hereditary information remains contentious [32–34]. This perspective heavily relies on the holobiont model of human-microbiome interactions. A holobiont refers to a host and its symbiotic microbes acting as a single evolutionary unit, and the combined pool of genetic material is referred to as the hologenome [35]. Taking the hologenome model to be correct, it has been implied that microbial genetic variation should be integrated into host phenotype heritability estimates [36–38].

**Fig. 1:**
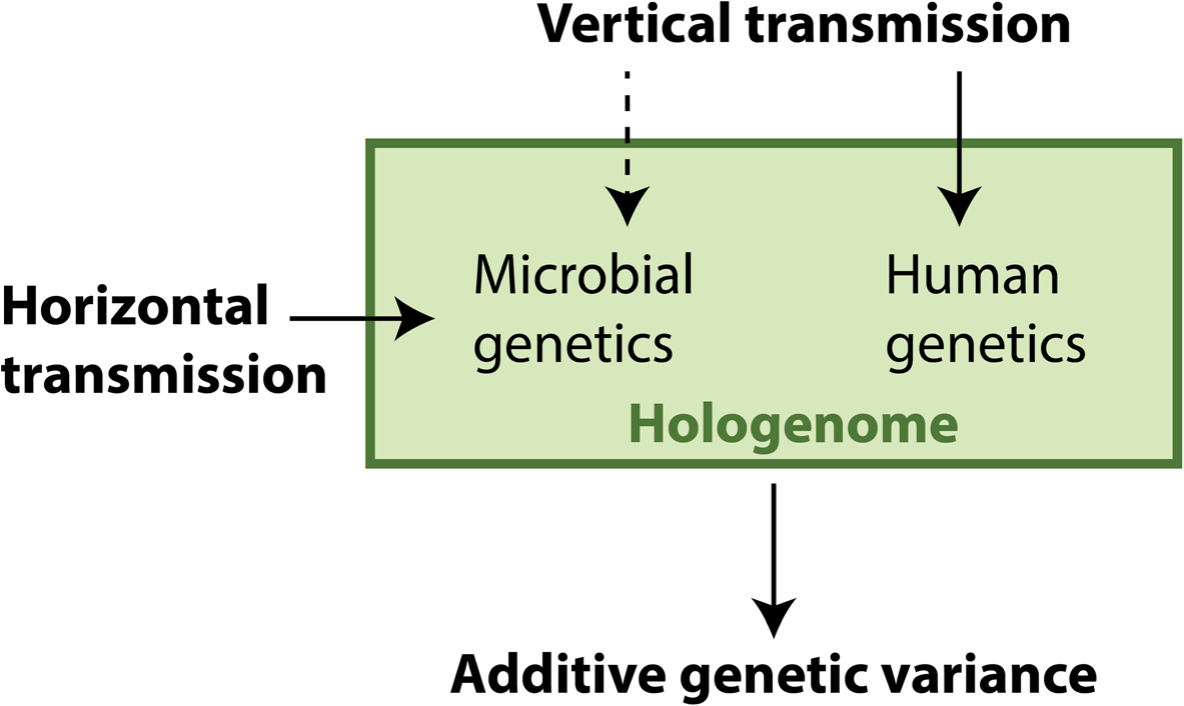
The key model that we argue against in this paper. Under this model, the genetic variations in both the human microbiome and genome are used to calculate the additive genetic variance component of narrow-sense heritability. In the strictest form of the model, microbial genetic variation can contribute to phenotype heritability even if it is horizontally transmitted (i.e., acquired from the environment). This is because the focus should be on the holobiont, the supraorganism of both microbiome and host, and its associated hologenome (the combined DNA of all constituents). The more lenient form of this model restricts the microbial genetic variation relevant to phenotype heritability to be the subset acquired through vertical transmission. The dotted arrow represents lower levels of vertical transmission of the microbiome relative to horizontal transmission

An explicit proposal for this perspective was outlined by Sandoval-Motta and colleagues [39] based on four observations: (1) the microbiome is associated with many traits and diseases, (2) the microbiome encodes a high number of genes, (3) human genotypes interact with the microbiome, but cannot account for most microbial variation, and (4) microbial genetic composition can be both horizontally and vertically transmitted. Based on these observations, the authors argued that trait heritability estimates inferred from classical heritability studies may be based on both human and microbial genetic variation. To address this issue, the authors suggested that the narrow-sense heritability of human phenotypes should be re-defined as the sum of the heritability derived from the effect sizes of additive human SNPs (${h}_{\mathrm{GWAS}}^2$) and the heritability estimated from microbial gene families $\left({h}_{\mathrm{MWAS}}^2\right):{h}^2={h}_{\mathrm{MWAS}}^2+{h}_{\mathrm{GWAS}}^2$. Although there are many technical challenges facing the implementation of this proposal, as the authors acknowledge, this model nonetheless represents a common perspective regarding microbial genetic variation [36–38] (Fig. 1). The four observations motivating this model are correct, and the first two observations are especially straight-forward. In particular, microbial variation has indeed been associated with myriad human traits and diseases [40]. In addition, there is an enormous degree of genetic variation within the microbiome, as described above.

Associations between the human genome and microbiome composition have also been extensively studied [41]. Several heritable taxa have been identified, meaning that a high proportion of the variability in relative abundance of these taxa across individuals is attributable to human genetic effects. One such taxon is the bacterial family Christensenellaceae, which has high heritability estimates ranging from 0.31 to 0.64, although its functional role within the gut is largely uncharacterized [42–44]. One functionally important heritable group is the archaeal family Methanobacteriaceae [42, 45], which reduces CO_2_ with H_2_ to methane in the gut.

Despite these examples of heritable microbial lineages, a recent analysis of two independent cohorts showed that variation in the overall composition of the gut microbiome is largely determined by environmental factors [46]. Although the central role of the environment in shaping the microbiome is widely appreciated [47], prior to this study the narrow-sense heritability of overall composition had not been directly quantified. To estimate this metric, the authors computed the heritability of each significantly heritable taxon weighted by its relative abundance in the TwinsUK dataset [42, 46]. Formally, community-wide ${h}^2={\sum}_{t\in S}{r}_t{h}_t^2$, where *S* is the set of significant taxa and ${r}_t\ \mathrm{and}\ {h}_t^2$ refer to the relative abundance and heritability of significant taxon *t*, respectively. The community-wide *h*^2^ was conservatively estimated to be 1.9% (and 8.1% when not performing multiple-test correcting while determining *S*). Importantly, this low estimate may change as additional data, such as inter-strain level variation, is integrated into community-wide *h*^2^ for other cohorts. However, based on the current data it appears that inter-individual variation in microbial composition is predominantly due to environmental effects.

This observation implies that most microbial genetic variation in the microbiome is a proxy for environmental, and not missing human genetic, variation. One possible counterargument might be that environmental factors affecting microbiome composition are irrelevant since this simply reflects the variable aspects of a holobiont’s identity. In this view, the holobiont is no less an individual than a human, which undergoes its own developmental and environmentally mediated changes through time. However, under the strict hologenome model, where the host and the microbiome act as a single evolutionary unit, there must be collective reproduction of the holobiont [33, 48]. Collective reproduction has direct relevance to its status as the “correct” level for assessing heritability, because that is the mechanism by which any biologically encoded information about phenotype is transmitted across generations. Because microbes are largely acquired horizontally and are influenced by myriad environmental factors, the pattern of vertical descent entailed by collective reproduction is largely broken, and so this strict form of the hologenome concept is invalid [33].

There are also operational difficulties with re-expressing heritability in terms of a hologenome. For instance, there is no single microbiome within humans: there are drastically different communities spatially and temporally [49, 50]. This is not analogous to the genetic variation between human cells in the body. The variant profiles used for GWASs are meant to approximate the original zygote genome in each individual, which does correspond to a single genome sequence. In contrast, there is no rationale for a microbiome sample from a single timepoint or body site to be specifically relevant to heritability. It would be possible to identify (albeit minor) variation in taxonomic and functional composition by profiling samples within 1 mm of each other. Researchers could thus theoretically produce thousands of microbiome profiles representing a single individual. There is likely no biologically satisfying way of integrating these profiles into a single measure of microbial genetic variance without additional information.

A stronger counterargument might be that a non-negligible proportion of microbial genetic variation should nonetheless be integrated into human trait heritability estimates based on a less strict form of the hologenome concept. It has been argued that although the hologenome model itself is flawed, the human holobiont could be considered in terms of functional interactions that can be performed by horizontally acquired microbes [48]. Under this model, different processes affecting microbial community assembly and stability lead to variation in the construction of functional niches within the human microbiome. These functional niches are filtered by natural selection such that niches which confer greater fitness are consistently re-constructed over evolutionary time. This explanation could account for heritable taxa that consistently fill a functional niche. It might be argued that the genomic variation of taxa filling a functional niche should be considered the same as different alleles at a locus in the human genome.

However, even under this more limited hologenome concept re-expressing narrow-sense heritability in terms of a hologenome remains problematic. While it may be useful to re-consider the microbiome in terms of functional niches that can be filled by different microbes [48], collective reproduction of the human host and members of each functional niche would nevertheless be required. The current evidence suggests that which microbes fill these niches is largely dependent on the environment, as discussed above, and so these niches would be highly sensitive to environmental contingencies. Due to this issue, this partial hologenome concept is unlikely to be relevant to human heritability. In addition, although human genetic variants might predispose individuals to certain colonizing microbes in a particular niche, this is subject to environmental exposure of those microbes. Such predisposing human genetic variants would already be included in heritability estimates, and the relevant microbial genetic variation would likely be represented by human GxE interactions (see next section).

A separate counterargument is based on evidence that certain microbes are transmitted between close relatives. In particular, mother-offspring microbial transmission is known to occur in humans during childbirth and early life [51]. A recent analysis showed that 16.4% of strains within infant microbiomes were shared with the respective mother, and these shared strains had higher gut colonization efficacies [52]. In contrast, only 0.73% of strains were shared between unrelated infants and mothers. At evolutionary timescales, there is also evidence of co-diversification of a small proportion of microbes across primate lineages [53], which on the surface is consistent with a more limited hologenome concept being relevant for human trait heritability calculations. These observations provide valuable biological insights, and it remains an exciting area of research to determine the degree to which vertically transmitted strains affect human phenotypes.

**Fig. 2:**
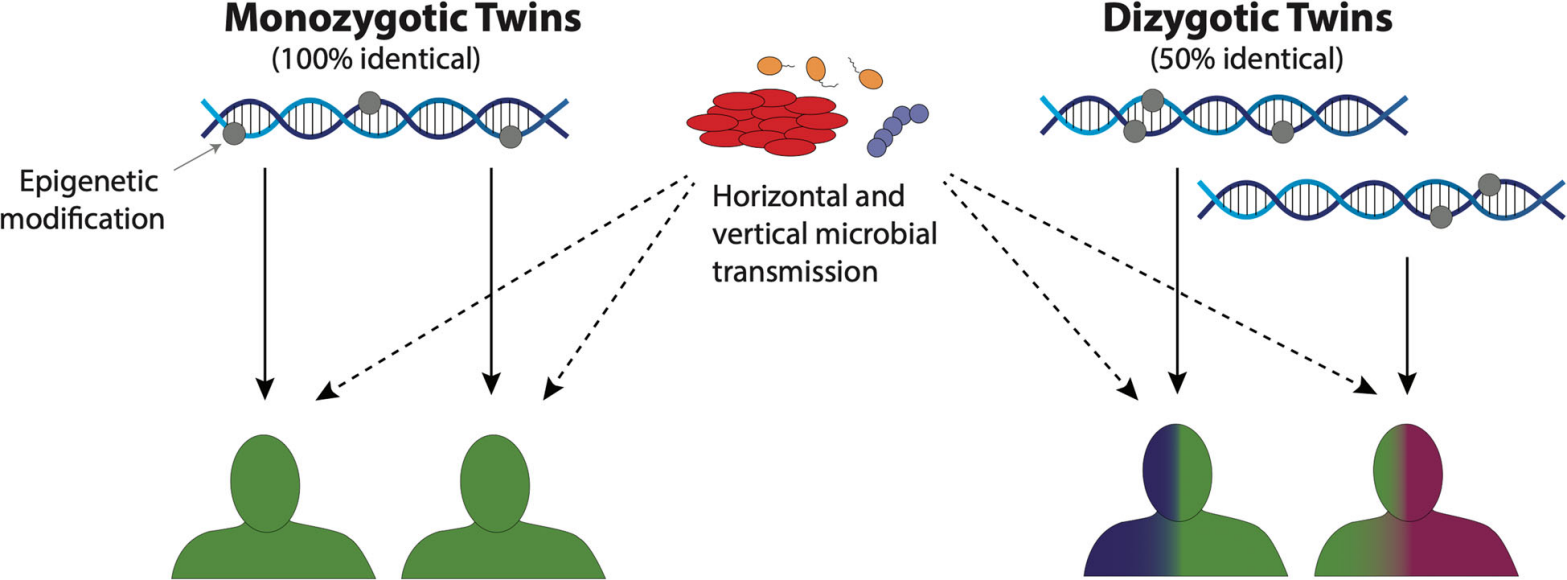
There is currently no clear rationale for why monozygotic twins would acquire more similar strains through vertical transmission compared to dizygotic twins in early life. This would be required for the vertical transmission of microbes to contribute to the problem of missing heritability, because traditional heritability estimates have been based predominately on comparing differences in phenotype concordance between monozygotic and dizygotic twins. A contrasting example is epigenetic signals (indicated by grey dots on DNA), which have also been suggested to be a partial explanation for the issue of missing heritability. Although this point remains highly controversial, the rationale is nonetheless much clearer because any such signals would be transmitted through the zygote genomes to each twin

Despite these observations, such strains are unlikely to make a substantial contribution to the high heritability estimates reported by classical studies. The majority of classical trait heritability estimates are derived from twin studies, which are based on comparing differences in phenotypic similarity between monozygotic twins and dizygotic twins [23, 54]. It is unclear what mechanism would result in higher concordance in the vertical transmission of strains between monozygotic twins compared to dizygotic twins (Fig. 2). This would be required for the microbiome to contribute to the high heritability estimates reported in classical studies. In contrast, there are clearer rationales for why other controversial potential contributors to missing heritability would be more similar in monozygotic twins. For example, epigenetic signals are physically linked to the human genome, and thus monozygotic twins could potentially acquire identical epigenetic signatures. Without a similar rationale for vertically transmitted strains, they are unlikely to be contributors to missing heritability.

Nonetheless, if a sufficient physical mechanism was identified, then vertically transmitted strains could contribute to the issue of missing heritability to some degree. The holobiont model would not be a requirement for this scenario, because the contribution of the microbiome to missing heritability would be a statistical phenomenon, rather than a missing source of human genetic variation. We anticipate three different requirements for this model: (1) emergence of heritable variance in the interactions between microbial and host genes, (2) emergence of interactions between the host and vertically transmitted microbes that increases the chance that children will resemble their parents, and (3) some degree of robustness to disruption by competition with horizontally acquired microbes. These requirements remain to be explored in the future to determine the viability of this model. However, if this model was shown to be reasonable, it would represent a distinct case from the main perspectives we have argued against in this work (Fig. 1). Indeed, such a model seems to capture a phenomenological effect similar to cases where the requirement for shared environmental effects across monozygotic and dizygotic twins is unmet [23, 24]. For example, dizygotic twins may be more likely to strive to differ from each other socially and cognitively compared to monozygotic twins [24]. Such unaccounted environmental and cultural factors do result in inflated estimates of human genetic effects in twin studies [23]. Similarly, the hypothetical higher concordance in strain transmission to monozygotic twins could result in inflated estimates of human genetic effects, and thus inflated heritability estimates. Therefore, such vertically transmitted strains could conceivably be related to the issue of missing heritability, although it would be incorrect to consider them a missing source of human genetic variation in GWASs.

## Leveraging the microbiome to detect gene-by-environment interactions

Based on our previous arguments, treating total microbial genetic variation as if it was a source of unmeasured human genetic variation is unjustifiable. However, microbiome sequencing data could nonetheless be a valuable datatype to integrate into GWAS frameworks. In the remainder of this paper, we describe several potential approaches for integrating microbiome data into such frameworks. Importantly, these approaches are not recommendations, but instead represent potentially useful additions to GWASs that remain to be evaluated.

Several recent studies have successfully integrated complementary biological datatypes into GWAS frameworks, which have focused on improving the functional interpretation of GWASs. For instance, epigenomics and chromosome conformation capture data were recently leveraged with known gene networks to more accurately identify candidate schizophrenia risk genes nearby significant GWAS loci [55]. Similarly, several biological datatypes, including tissue-specific transcriptomics, epigenomics, and genome-wide SNPs, were integrated to identify putative regulatory networks and pathways underlying psoriasis genetic risk [56]. Integrating microbiome data into similar systems biology frameworks might yield more interpretative insights from GWASs in the future. However, in general this approach is likely unfeasible currently due to the high variability and relatively poor mechanistic characterization of the microbiome.

A different approach to consider is to use microbiome profiles (i.e., taxonomic and functional relative abundances) as a proxy for how environmental factors differ across individuals in a cohort to better control for GxE interactions. Several “omics” datatypes have previously been suggested as potential means of measuring environmental exposures, including the microbiome [19, 20]. However, which collection of datatypes would be optimal and how to best integrate this information into existing GWASs and GxE association frameworks remains unclear.

Nonetheless, recent studies of the human microbiome highlight that this datatype would be informative for capturing environmental variation across cohorts. For instance, it was recently shown that Thai immigrants to the USA quickly acquire a microbial signature that becomes more pronounced with increased duration of stay [57]. This signature includes an overall drop in alpha-diversity as well as the loss of several taxa. The ratio of *Bacteroides* to *Prevotella* relative abundances showed particularly pronounced changes and was positively associated with duration of stay. Interestingly, dietary differences could only partially account for these differences, suggesting that other unmeasured environmental factors are being captured by such microbial profiles. Nonetheless, diet is typically the most easily attributable factor affecting microbiome variation. This fact is reflected by the observation that dietary metrics, such as the number of different plants consumed, were identified as major drivers of microbiome variation in the American Gut cohort [58].

These recent examples imply that the major axes of variation in the microbiome partially represent cultural variation, which itself partially represents differential exposures to environmental factors. Stratifying a cohort by these axes into discrete groups could be one approach to leverage this information. For instance, if individuals could be clearly clustered into groups based on their microbiome profiles, it might be appropriate to test for significant genetic variants for each group separately. This approach would be analogous to stratifying populations by sex, which has resulted in uncovering genetic variants of interest that were obscured when both sexes were analyzed together [59]. A major drawback of unsupervised clustering of a population into groups based on the overall microbiome profile is that results could be difficult to generalize unless the involved microbiome clusters just happened to be predictive of an environmental covariate.

Another potential approach for integrating microbiome data into GWAS frameworks would be to correct for environmental variation between individuals. This approach would be similar to controlling for variation in shared ancestry across a cohort. It is common to control for population stratification among GWAS participants by separately correcting for the major axes of variation derived from a principal components analysis computed based on the genetic distance between participants [60]. The major axes of variation derived from multidimensional scaling of microbiome relative abundance data could be used analogously to control for environmental variation across individuals. The major drawback of this approach would be that human genetic effects could be obscured if variation in the microbiome was strongly associated with trait values. For instance, if antibiotic usage were higher for disease cases than controls, then the major axes of microbial variation would likely be directly related to disease state. In addition, the above approach could reduce statistical power in case-control studies by reducing the precision of effect estimates for diseases with low prevalence [61].

Fortunately, these are not novel issues for GWASs since there are often clinical phenotypes measured that are related to disease liability or other focal clinical phenotypes. For example, body mass index is an important covariate of waist to hip ratio and waist circumference. GWASs focused on these two waist phenotypes that include body mass index as a covariate can result in biased genetic variant effect estimates [62]. For case-control studies, one way to circumvent this problem is to condition on covariates in a liability model that incorporates external information about the covariate [63]. This approach is called informed conditioning and enables disease liability to be modelled as a function of a covariate and published prevalence data for the covariate. For instance, age is an important covariate for prostate cancer since prevalence is 2%, 8%, and 14% for men aged 60, 70, and 80, respectively [63]. Explicitly including this information in a liability model of prostate cancer was shown to increase statistical power. Including reproducible microbiome signatures of environmental factors (e.g., diet) in such models might similarly increase statistical power. Although this approach seems promising, several problems remain (1) the subjective and potentially incomplete summary provided by microbial features to represent an environmental factor, (2) the potential for confounding among large numbers of environmental covariates, and (3) microbiomes can be extremely complex, and we often have little prior knowledge of which features are associated with different aspects of the environment. Future work is needed to address these problems and to assess the feasibility of integrating microbiome sequencing data into informed conditioning models and GWASs in general.

## Conclusions

There are multiple fundamental issues with considering the microbiome as an extension of the human genome. Accordingly, non-microbial explanations for missing heritability, such as the presence of many low-effect human genetic variants, are more plausible than that microbial genetic variation is a missing source of human genetic variation. Nonetheless, microbiome sequencing data could still potentially be useful for addressing missing heritability by instead controlling for confounding environmental variation in GWASs. Although several approaches appear promising, the feasibility and benefits of leveraging microbiome data as a proxy for environmental variation in GWASs remain to be determined.

## Data Availability

Not applicable.
